# Comparison of two different frailty screening scales for predicting mortality due to all causes in older inpatients

**DOI:** 10.1590/1806-9282.20240250

**Published:** 2024-08-16

**Authors:** Meris Esra Bozkurt, Tugba Erdogan, Cihan Kilic, Humeyra Ozalp, Gulcin Ozalp, Emine Asci, Zeynep Fetullahoglu, Caglar Ozer Aydın, Gulistan Bahat, Mehmet Akif Karan

**Affiliations:** 1 Mersin City Hospital, Department of Internal Medicine, Division of Geriatrics - Mersin, Turkey.; 2 Istanbul University, Istanbul Medical School, Department of Internal Medicine, Division of Geriatrics - İstanbul, Turkey.; 3 Goztepe Prof. Dr. Suleyman Yalcin City Hospital, Department of Internal Medicine, Division of Geriatrics - İstanbul, Turkey.

**Keywords:** Frailty, Mortality, Comparison, Older adults

## Abstract

**OBJECTIVE:**

This study examines the relationship between two frailty screening tools and 90-day all-cause mortality in geriatric inpatients.

**METHODS:**

The study included patients aged ≥60 years who were admitted to the geriatrics unit of a university hospital between June 2021 and August 2022 and whose mortality status and duration of hospitalization data were obtained from the Health Ministry System. During hospitalization, the patients were screened using two different frailty scales: the Simpler Modified Fried Frailty Scale (sMFS) and the Clinical Frailty Scale (CFS). Patients scoring ≥5 on the CFS and ≥3 on the sMFS were considered frail.

**RESULTS:**

A total of 84 participants with a mean age of 78.3±7.6 years were included in this study, of which 36.9% were male. Of the total, 60.7% and 89.3% were considered frail according to the CFS and sMFS, respectively, and the prevalence of all-cause mortality within 90 days was 19%. A univariate analysis using the Kaplan-Meier survival method revealed CFS scores to be statistically significantly related to 90-day all-cause mortality (p<0.001), while sMFS scores were not found to be statistically significant (p=0.849). Furthermore, a statistically significant relationship was identified between CFS score and all-cause mortality in multivariate analysis with Cox regression analysis [(p<0.001), hazard ratio (HR): 3.078; (95% confidence interval: 1.746-5.425)].

**CONCLUSION:**

An evaluation of frailty in hospitalized older adults using two different scales revealed the CFS to be superior to the sMFS in predicting all-cause mortality within 90 days.

## INTRODUCTION

The general health status of older adults can range from completely healthy and independent in daily activities to being completely dependent and bedridden^1,2^. The approaches to the health management of older adults and the potentially wide spectrum of health conditions that may be encountered thus necessitate different approaches^1,2^. Overdiagnosis in older adults whose mortality is expected within a few months should be avoidable through scientific approaches. This has led to the definition of the concept of "frailty"^1,2^. Frail older adults are the most complex patient group to follow up due to the difficulties encountered in the management of chronic diseases, the different treatment goals, and the presence of multiple comorbidities and their associated problems^3^. The frailty concept is adopted to identify people at greater risk of adverse health outcomes associated with, for example, falls, recurrent hospitalizations, placement in a nursing home, dependency, and mortality^1-4^.

Numerous frailty screening tools have been developed for the assessment of frailty, such as the Fried Physical Frailty Scale, the Frailty Index, the Rockwood Clinical Frailty Scale (CFS), and the FRAIL Scale^1^. The Fried Physical Frailty Scale was one of the first such assessment scales to be introduced to the field^3,5-7^. The Fried Scale is based on a formal and detailed assessment of the patient's self-reported kilocalorie per week expenditure^5,7^. However, This assessment approach takes a lot of time and relies on the cognitive function of the responding older adult. Likewise, hand grip strength requires evaluation with a hydraulic hand dynamometer, while walking speed is a formal evaluation measured in meters/second. For all the above reasons, the Fried Frailty Scale can be considered an impractical screening tool for use in clinical practice as most patients hospitalized in geriatric units are unable to stand or may have sequelae symptoms^8^.

Based on an evaluation of responses to five questions asked to the patient or close caregiver, frailty determined using the Simplified Modified Fried Frailty Scale (sMFS) has been shown to predict mortality in nursing home residents^5^. The five questions relate to involuntary weight loss, feelings of exhaustion-burnout, weakness (hand grip strength), slow walking speed, and low physical activity^5,6^.

The Clinical Frailty Scale (CFS), which was created by Rockwood et al., is based on the observation of the patient by a physician^9^. It is a practical tool for frailty screening in geriatric service settings, where care plans for older adults with multiple problems need to be devised quickly, due to the easy applicability of the nine items^9^.

The intention of this study is to meet a need of physicians in the geriatric care of older adults, the management of which can be difficult and complex, through the identification of the optimum frailty screening tool in terms of speed, ease of application, and ability to predict mortality. To this end, we compare the ability of the sMFS and the CFS to predict all-cause mortality within 90 days of discharge.

## METHODS

Patients aged 60 years and over who were admitted to the geriatric service between June 2021 and August 2022 were included in this retrospective study. Prior to hospitalization, all patients were tested for COVID-19 in the emergency room or COVID-19 polyclinics, and those with positive or suspected COVID-19 results were admitted to separate COVID-19 services. Patients who died during hospitalization, those whose hospitalization continued, and those who were transferred to surgical services were excluded from the study. Approval for the study was obtained from the ethics committee of a local university (Reference number/1083), and all procedures in the study were carried out in accordance with the principles defined by the Declaration of Helsinki. The data of all participants admitted to the geriatric service were recorded by a responsible internal medicine doctor other than the attending physician. All information was obtained within the first 2 days of the patient's admission. The demographic characteristics of the patients (age, gender), presence of chronic diseases, activities of daily living (ADL), instrumental activities of daily living (IADL), presence of geriatric syndromes (falls, frailty, malnutrition, urinary incontinence, sleep disorders), presence of frailty fracture in the last 1 year, and duration of hospitalization were recorded. The risk of malnutrition was assessed using the Mini Nutritional Test-Short Form (MNA-SF). Patients with an MNA-SF score of <11 were considered at risk of malnutrition^10^. Polypharmacy was identified as four drugs^11^.

Frailty screening using the sMFS is based on an evaluation of the responses to five questions asked to the patient or close caregiver^5^. The five questions are related to involuntary weight loss, fatigue/feelings of burnout, weakness (hand grip strength), slow walking speed, and low physical activity^5,6^. In this study, patients scoring ≥3 on the sMFS scale were evaluated as frail.

The CFS is based on physician observations, for which patients are evaluated on a scale of 1-9 in which 1 indicates very fit and 9 indicates terminally ill^9^. Patients scoring ≥5 on the CFS scale are considered frail^9^. Details of the patients who were discharged from the hospital were obtained from the hospital records.

### Statistical analysis

Following the evaluation of data distribution using a Kolmogorov-Smirnov test. The association between the data distribution by gender and the other study variables was evaluated with chi-square, Mann-Whitney U, and independent-sample t-tests, depending on the characteristic properties of the data. The Kaplan-Meier method was used to evaluate the relationships between the length of stay, 90-day all-cause mortality data, and frailty scales. The Cox regression model was used to examine the associations between the frailty scales and 90-day all-cause mortality, for which hazard ratios (HRs) with 95% confidence intervals (CIs) were calculated.

## RESULTS

A total of 84 participants with a mean age of 78.3±7.6 years were included in this study, of which 36.9% were male. The application of the CFS and sMFS revealed 60.7 and 89.3% of the sample to be frail, respectively, and all-cause mortality within 90 days was 19%. The median length of hospitalization of the study population was 15.5 (1-158) days.

In univariate analyses, age, presence of dementia as a chronic disease, urinary incontinence, number of chronic diseases, number of chronic drugs, ADL, IADL, length of stay, and mortality were all identified as statistically significant factors, as identified by the CFS frailty screening tool (p-values, respectively, p=0.001, p=0.002, p=0.007, p <0.001, p=0.017, p <0.001, p<0.001, p<0.001, and p<0.001) (Table 1).

**Table 1:** Relationship between CFS frailty screening scale in univariate analyses with demographic characteristics, chronic diseases, geriatric syndromes, 90-day all-cause mortality, and length of stay.

		CFS≥5 (n=51) 60.7%	CFS<5 (n=33) 39.3%	Total (n=84) 100%	p-value
Age		80.4± 6.8	75 ±7.7	78.3±7.6	0.001Θ
Gender					
	Male	17 (33.3%)	14 (42.4%)	31 (36.9%)	0.399
	Female	34 (66.7%)	19 (57.6%)	53 (63.1%)	
Chronic disease (n, %)					
	CHF	16 (31.4%)	7 (21.2%)	23 (27.4%)	0.308
	CKF	10 (19.6%)	9 (27.3%)	19 (22.6%)	0.412
	COPD	6 (11.8%)	3 (9.1%)	9 (10.7%)	0.699
	DM	23 (45.1%)	12 (36.4%)	35 (41.7%)	0.428
	Dementia	18 (35.3%)	2 (6.1%)	20 (23.8%)	0.002Θ
	Depression	11 (21.6%)	3 (9.1%)	14 (16.7%)	0.134
	HT	40 (78.4%)	23 (69.7%)	63 (75%)	0.367
Geriatric syndromes (n, %)					
	Falls	28 (54.9%)	17 (51.5%)	45 (53.6%)	0.761
	Undernutrition (MN+MNR)┴	48 (96%)	33 (100%)	81 (97.6%)	0.245
	Frailty fracture in last year┴	8 (15.7%)	3 (9.1%)	11 (13.3%)	0.409
	Urinary incontinence	44 (86.3%)	20 (60.6%)	64 (76.2%)	0.007Θ
	Sleep disorders×	32 (64%)	17 (53.1%)	49 (59.8%)	0.327
	Polypharmacy (n, %)	50 (98%)	29 (87.9%)	79 (94%)	0.055
	Number of chronic drugs	11.3 ±3.5	7.9± 3.5	10±3.9	<0.001Θ
	Number of chronic diseases	4.8 ±2.3	3.7±1.5	4.3±2.1	0.017Θ
	ADL*	1 (0-6)	6 (5-6)	5 (0-6)	<0.001Θ
	IADL*	1 (0-8)	8 (3-8)	4 (0-8)	<0.001^Θ^
	Length of stay*	18 (4-158)	12 (1-50)	15.5 (1-158)	<0.001Θ
	90-day mortality (days)	11 (21.6%)	5 (15.2%)	16 (19%)	<0.001Θ

ADL: activities of daily living

CHF: congestive heart failure

CKF: chronic kidney failure

COPD: chronic obstructive pulmonary disease

DM: diabetes mellitus

HT: hypertension

CFS: Clinical Frailty Scale

IADL: instrumental activities of daily living

MN: malnutrition

MNR: malnutrition risk

* Given data as median.

Θ Significant p-values.

┴ Marked data include 83 participants.

× marked data include 82 participants.

In the univariate analyses, the factors associated with the sMFS frailty screening tool were determined as age, ADL, and IADL (p=0.004, p=0.008, and p<0.001, respectively) (Table 2).

**Table 2:** Relationship between sMFS frailty screening scale in univariate analyses with demographic characteristics, chronic diseases, geriatric syndromes, 90-day all-cause mortality, and length of stay.

		sMFS≥3 (n=75) 89.3 %	sMFS<3 (n=9) 10.7%	Total (n=84) 100%	p-value
Age		79.2 ±7.5	71.5 ± 5.1	78.3±7.6	0.004Θ
Gender					
	Male	25 (33.3%)	6 (66.7%)	31 (36.9%)	0.050
	Female	50 (66.7%)	3 (33.3%)	53 (63.1%)	
Chronic disease (n, %)					
	CHF	22 (29.3%)	1 (11.1%)	23 (27.4%)	0.247
	CKF	14 (18.7%)	5 (55.6%)	19 (22.6%)	0.012Θ
	COPD	8 (10.7%)	1 (11.1%)	9 (10.7%)	0.968
	DM	32 (42.7%)	3 (33.3%)	35 (41.7%)	0.592
	Dementia	20 (26.7%)	0 (0%)	20 (23.8%)	0.076
	Depression	14 (18.7%)	0 (0%)	14 (16.7%)	0.156
	HT	57 (76%)	6 (66.7%)	63 (75%)	0.541
Geriatric syndromes (n, %)					
	Falls	42 (56%)	3 (33.3%)	45 (53.6%)	0.198
	Undernutrition (MN+MNR)┴	72 (97.3%)	9 (100%)	81 (97.6%)	0.618
	Frailty fracture in last year┴	10 (13.5%)	1 (11.1%)	11 (13.3%)	0.841
	Urinary incontinence	56 (74.7%)	8 (88.9%)	64 (76.2%)	0.344
	Sleep disorders×	44 (58.7%)	5 (55.6%)	49 (59.8%)	0.785
	Polypharmacy (n, %)	71 (94.7%)	8 (88.9%)	79 (94%)	0.489
	Number of chronic drugs	10.2 ±3.8	7.6 ±3.7	10±3.9	0.058
	Number of chronic diseases	4.4±2.2	3.7± 1.5	4.3±2.1	0.367
	ADL*	5 (0-6)	6 (5-6)	5 (0-6)	0.008^Θ^
	IADL*	3 (0-8)	8 (7-8)	4 (0-8)	<0.001Θ
	Length of stay*	15 (1-158)	16 (4-50)	15.5 (1-158)	0.862
	90-day mortality (days)	16 (21.3%)	0 (0%)	16 (19%)	0.849

ADL: activities of daily living

CHF: congestive heart failure

CKF: chronic kidney failure

COPD: chronic obstructive pulmonary disease

DM: diabetes mellitus

HT: hypertension

IADL: ınstrumental activities of daily living

MFS: Simpler Modified Fried Scale

MN: malnutrition

MNR: malnutrition risk

* Given data as median.

Θ Significant p-values.

┴ Marked data include 83 participants.

× Marked data include 82 participants.

A statistically significant correlation was found between the CFS and sMFS frailty screening tools (r=0.602, p<0.001).

In univariate analyses using the Kaplan-Meier survival method, the CFS was statistically significantly related to 90-day all-cause mortality (p<0.001), while in univariate analyses of the sMFS, it was found not to be statistically significant (p=0.849). A statistically significant relationship was revealed between the CFS and all-cause mortality after adjusting for age, sex, undernutrition, number of diseases, and falls in the evaluation of the screening tools in multivariate analysis with Cox regression analysis [(p<0.001), HR: 3.078; 95%CI: 1.746-5.425]. Although not significant in the univariate analyses, the sMFS tool and all-cause mortality association remained statistically insignificant after adjusting for age, sex, malnutrition risk, number of drugs, and falls (Table 3).

**Table 3:** Relationship of CFS and sMFS screening tools with 90-day all-cause mortality after adjustment in Cox regression.

		Age	Gender	Undernutrition risk┴	Number of chronic diseases	Dementia	Falls	CFS≥5
p		0.062	0.558	1	0.222	0.743	0.854	<0.001Θ
HR		1.033	0.867	1	0.932	1.115	1.047	3.078
95%CI								
	-Lower	0.998	0.539	0.209	0.832	0.582	0.641	1.746
	-Upper	1.069	1.396	4.790	1.044	2.134	1.710	5.425
		Age	Gender	Undernutrition risk┴	Number of chronic diseases	Dementia	Falls	MFS≥3
p		0.609	0.588	0.713	0.366	0.312	0.756	0.900
HR		1.008	0.877	0.749	0.950	1.376	1.079	1.050
95%CI								
	-Lower	0.976	0.547	0.161	0.849	0.741	0.667	0.491
	-Upper	1.042	1.408	3.490	1.062	2.557	1.745	2.246

CFS: Clinical Frailty Scale

CI: confidence ınterval

HR: hazard ratio

MFS: Simpler Modified Fried Scale.

Θ Significant p-values.

┴ Marked data include 83 participants.

## DISCUSSION

This study of 84 geriatric service patients aged 60 years and older assessed and compared the capacity of the CFS and sMFS frailty scales to predict 90-day all-cause mortality. A moderate correlation was noted between the CFS and sMFS in the results of the study; although a significant statistical relationship was noticed between all-cause mortality and CFS, no such relationship was identified with the sMFS. Neither the CFS nor sMFS results showed statistical significance with duration of hospitalization.

Frailty was identified in 60.7% of the geriatric inpatients based on the CFS, while the sMFS identified 89.3% of the participants as frail. All-cause mortality within 90 days was 19%.

There are studies comparing the ability of various frailty scales to predict mortality in different patient groups. In a study comparing the ability of the Fried Scale and the CFS to predict 90-day mortality among the older adults admitted to the emergency department, an association was identified between mortality and CFS, concurring with the results of the present study^12^, which may be due to the similarity of the patient population. In addition, as symptoms such as decreased physical activity and fatigue may be common in all patients with acute illnesses, the effectiveness of the Fried scale or the scales derived from it may be limited. Future studies may come up with revisions to the sMFS allowing its application in acute situations. In a further study assessing the ability of the FRAIL Scale, Fried Scale, and CFS to predict 28-day mortality and re-hospitalization in emergency older patients, none of the scales was found to predict rehospitalization, while all three were able to predict mortality, with the predictions based on the Fried Scale being more accurate^13^. These results conflict with the findings of the present study, which may be attributable to the different accompanying comorbidities of the patients, the level of objectivity of the responses of patients or their relatives, and the more objective nature of the present study due to the observations being made by a single physician.

There are also studies of patients undergoing geriatric rehabilitation comparing the ability of different frailty scales to predict adverse clinical outcomes^14,15^. In a study by Soh et al. of patients undergoing geriatric rehabilitation, the ability of the Frailty index laboratory, modified Frailty index laboratory, and CFSs to predict 1-year mortality was evaluated^14^, and the authors found all three scales to be poor predictors of mortality in elderly patients undergoing geriatric rehabilitation^14^. This difference may be due to the longer mortality period assessed in this study and the different patient populations.

Bahat et al. found that sMFS was able to predict mortality after 4 years in their study of 224 nursing home residents to a statistically significant degree^5^. While the validity and reliability of the sMFS have been established^6^, studies comparing the relationship with predicting mortality of sMFS in geriatric service patients with other studies are not yet available in the literature.

In a prospective study comparing four different scales, namely, the FRAIL, the Tilburg Frailty Indicator, the CFS, and the Frailty Index, in terms of their ability to predict loss of functionality, institutionalization, length of hospital stay, and mortality during hospitalization in the geriatric patient population, CFS was found to better predict loss of functionality and length of stay^16^. These results differ from those of our study in two ways. First, the ability of the scales to predict loss of functionality was not assessed in this study, which may be due to the difference in the definition of mortality, and second, the duration of hospitalization differed from those reported in this study, which may be due to the difference in the definition of frailty.

## CONCLUSION

This is the first study to compare the ability of the sMFS and CFS to predict 90-day all-cause mortality in a hospitalized geriatric patient population. The CFS was found to predict mortality in geriatric hospitalized patients, and the results reveal that physician observations are more consistent than those reported by the patients and their relatives. Further observational prospective studies are required to assess the ability of the sMFS to predict adverse clinical outcomes in geriatric inpatients.

## ETHICS APPROVAL

We obtained ethical approval from the ethical board of Istanbul University Medical School (Approval number: 2023/1083).

## CONSENT TO PARTICIPATE

We received informed consent from all participants.

## CONSENT FOR PUBLICATION

We received informed consent from all participants.
